# Using exercises to improve public health preparedness in Asia, the Middle East and Africa

**DOI:** 10.1186/1756-0500-7-474

**Published:** 2014-07-27

**Authors:** David J Dausey, Melinda Moore

**Affiliations:** 1 School of Health Professions and Public Health, Mercyhurst University, Erie, PA, USA; 2 Heinz College, Carnegie Mellon University, Pittsburgh, PA, USA; 3 Health Unit, RAND Corporation, Arlington, VA, USA

**Keywords:** Tabletop exercises, Public health preparedness, Regional, Sub-regional, Network, Public health, Global health

## Abstract

**Background:**

Exercises are increasingly common tools used by the health sector and other sectors to evaluate their preparedness to respond to public health threats. Exercises provide an opportunity for multiple sectors to practice, test and evaluate their response to all types of public health emergencies. The information from these exercises can be used to refine and improve preparedness plans. There is a growing body of literature about the use of exercises among local, state and federal public health agencies in the United States. There is much less information about the use of exercises among public health agencies in other countries and the use of exercises that involve multiple countries.

**Results:**

We developed and conducted 12 exercises (four sub-national, five national, three sub-regional) from August 2006 through December 2008. These 12 exercises included 558 participants (average 47) and 137 observers (average 11) from 14 countries. Participants consistently rated the overall quality of the exercises as very good or excellent. They rated the exercises lowest on their ability to identifying key gaps in performance. The vast majority of participants noted that they would use the information they gained at the exercise to improve their organization’s preparedness to respond to an influenza pandemic. Participants felt the exercises were particularly good at raising awareness and understanding about public health threats, assisting in evaluating plans and identifying priorities for improvement, and building relationships that strengthen preparedness and response across sectors and across countries. Participants left the exercises with specific ideas about the most important actions that they should engage in after the exercise such as improved planning coordination across sectors and countries and better training of health workers and response personnel.

**Conclusions:**

These experiences suggest that exercises can be a valuable, low-burden tool to improve emergency preparedness and response in countries around the world. They also demonstrate that countries can work together to develop and conduct successful exercises designed to improve regional preparedness to public health threats. The development of standardized evaluation methods for exercises may be an additional tool to help focus the actions to be taken as a result of the exercise and to improve future exercises. Exercises show great promise as tools to improve public health preparedness across sectors and countries.

## Background

Since 2001, there has been a dramatic increase in the use of disaster preparedness exercises among public health agencies in the United States [1,2]. These exercises have explored a wide range of topics from foodborne toxoplasmosis outbreaks [3], chemical disasters [4], acute blood shortages [5], bioterrorism [6], and severe acute respiratory syndrome (SARS) [7]. Exercises have been designed to assess and improve a variety of capabilities such as regional disaster preparedness among rural hospitals [8], knowledge and confidence of legal authorities [9], resource allocation [10] and risk communications [11]. A large number of these exercises have been focused on the spread of infectious diseases especially the threat of pandemic influenza because the common challenges pandemic influenza shares with other types of public health emergencies [12-16].

Our knowledge of the use of exercises for public health-related disaster preparedness outside the United States is much more limited. A considerable number of these types of exercises in the United States have been published in the academic literature but few findings from exercises that have taken place outside the United States have been published. Some researchers in the United States have tried to solve this gap by publishing the findings of “virtual” internet-based, long distance exercises conducted remotely with international partners [17,18]. Even less is known from direct experiences with in-country exercises or exercises that span multiple countries in a given region. The results of these types of exercises may be reported directly to exercise participants but often don’t make it to the scientific literature. If the results from exercises are published in any systematic way, they often get published in in-house publications for domestic audiences rather than scientific journals with a more global reach. The incentives, financial or otherwise, for researchers to turn these in-house publications into scientific papers are limited. This is a major loss to our knowledge base because countries around the world are increasingly recognizing the importance of transnational efforts to complement national efforts to detect and respond to public health threats quickly and effectively [19-23]. Exercises provide these countries with a vehicle to collaborate and test their ability to respond to these transnational threats. Exercises also provide these countries with an avenue to build relationships and trust among colleagues across sectors and across borders [24].

## Methods

We developed pandemic influenza tabletop exercises that built on the “Day After” methodology developed by Millot, Molander and Wilson [25] and described elsewhere in greater detail [1]. Countries in three different geographic regions participated: Southeast Asia (Cambodia, China, Lao PDR, Myanmar, Thailand and Vietnam), the Middle East (Israel, Jordan and Palestine) and East Africa (Burundi, Kenya, Rwanda, Tanzania and Uganda). Countries that participated in the exercises were included because they were all part of sub-regional disease surveillance networks established in part through funding from the Rockefeller Foundation. Some countries not included in these networks were invited to observe the exercises. Countries ranged in their past experience with preparedness exercises with some countries having extensive past exercise experience (such as Israel, Vietnam, China and Thailand) and other countries having minimal past exercise experience (such as Cambodia, Lao PDR, Myanmar and Uganda). Exercises were developed and conducted by exercise planning teams that included external exercise development experts from the RAND Corporation as well as senior health leaders from each of the respective localities and/or countries represented in the exercise. There were three different levels of exercises: sub-national (e.g., one or more provincial areas), national (e.g., one country) and sub-regional (e.g., multiple countries from one geographic region).

All exercises were multi-sectorial in nature meaning that they involved representatives from more than one sector of government. Examples of sectors included were health, agriculture, defense and environment. Each exercise focused on three to six different broad topic areas such as surveillance and information sharing, disease control, and communications that were identified in previous exercises as important [2,12]. Because Thailand had considerable previous experience with exercises, it designed and conducted its sub-national exercise with limited involvement from representatives at RAND. Exercise discussions focused on one topic area at a time each lasting from 30 to 90 minutes. Participants of exercises were selected by the exercise planning team and differed from exercise to exercise, but all exercises included representatives from the health sector of the locality and/or country represented. They also included senior leaders from at least one other non-health sector. In addition to participants, exercises also had “observers” who were invited to watch the exercise but did not directly engage in exercise discussions. All exercises were led by one or two exercise “facilitators” who directed the exercise discussion and probed participants for more information. In general, exercise facilitators can represent a range of disciplines from media professionals to health professionals. In these exercises the facilitators were all health officials or health researchers who were trained in the facilitation of exercises and who had extensive experience facilitating past exercises.

Exercises presented participants with a future scenario that involved an unfolding pandemic influenza crisis at different stages. They were required to respond to the scenario with the actions they would take if the scenario were actually occurring. Exercise facilitators were given discussion points and probes to keep the discussion focused and moving forward. Each section of the exercise ended with participants being asked to make concrete decisions for the topic area being discussed before moving on. The exercise concluded with a debriefing in which all participants evaluated their own response in light of what they learned during the exercise.

All exercise participants were asked to complete an evaluation form immediately after the exercise, before leaving the room. Typically, they spent about 15 minutes to respond to these questions. Six of the exercises were rated for their quality through five Likert scale questions: the overall quality of the exercise (1 = poor; 5 = excellent); the quality of the exercise discussions (1 = poor; 5 = excellent); the exercise identified important key gaps in preparedness (1 = strongly disagree; 5 = strongly agree); the exercise helped participants to better understand the roles and responsibilities of agencies and organizations responding to an influenza pandemic (1 = strongly disagree; 5 = strongly agree); and the exercise generated information that participants planned to use (1 = strongly disagree; 5 = strongly agree).

The remaining six exercises asked participants three different qualitative questions: what was the importance of the exercise; what are the most important actions that should be taken based on the exercise; and what suggestions do you have to help improve future exercises. In addition to participant evaluations, detailed After Action Reports (AARs) were developed for each exercise that summarized the exercise discussions and highlighted key aspects of each exercise. In January 2013, health leaders who were involved in the planning of the exercises from a subset of countries participated in brief semi-structured face-to-face interviews to discuss how their country followed up with the exercises and the current state of their exercise program. Health leaders included health officials working for the ministry of health in their respective countries. These health leaders were all directors of departments (such as communicable disease) within their ministry.

## Results and discussion

We developed and conducted 12 exercises from August 2006 through December 2008: four sub-national exercises, five national exercises, and three sub-regional exercises (Table 1). Across all of these exercises there were a total of 558 participants and 137 observers from 14 countries. The average number of participants per exercise was 47 and the average number of observers was 11. Participants from the health sector were represented in every exercise. The most commonly represented sectors other than health were agriculture and defense. Four exercises were shorter than one full 8-hour day in length, three exercises were one full day in length, and five exercises were more than one full day in length. The average length of the exercises was 9.75 hours. All exercises covered three to six of the topic areas outlined in Table 2.

**Table 1:** Characteristics of exercises

**Location**	**Type**	**Date(s)**	**Length in hours**	**# Participants (# Observers)**	**Sectors participating**
Cambodia	Sub-national	6 Sep 2006	8.0	50 (17)	Health, defense, commerce, information
China	Sub-national	27 Sep 2006	7.0	28 (7)	Health, emergency response, agriculture, border control
Laos	National	11 Oct 2006	8.0	37 (16)	Health, information and culture, agriculture and forestry, foreign affairs
Myanmar	National	16 Oct 2006	6.5	32 (10)	Health, port, veterinary
Thailand	Sub-national	25 Aug 2006	6.5	102 (30)	Health, agriculture
Vietnam	National	19 Oct 2006	4.0	38 (11)	Health, agriculture, rural development, defense, police, finance, transport, planning and investment
Palestine	National	26 Oct 2007	8.0	29 (0)	Health, UNFPA, UNRWA, UNDP, UNICEF
Jordan	Sub-national	8-9 Jan 2008	10.0	61 (5)	Health, civil defense, environment, police, public works, religious, education, social development, agriculture, youth, water
Jordan	National	9-10 Apr 2008	15.5	55 (5)	Health, agriculture, water and irrigation, energy, labor, finance, municipal and rural affairs, industry and trade, religious, education, justice, interior, transportation, maritime, civil aviation, media
Southeast Asia	Sub-regional	13-14 Mar 2007	16.5	59 (25)	Health, livestock and fishery, agriculture and forestry, communication and transport, immigration, security, tourism, information and culture, justice, finance, policy and planning, defense
Middle East	Sub-regional	30-31 Aug 2008	15.0	32 (3)	Health, information, international relations, industry-trade
East Africa	Sub-regional	16-17 Dec 2008	12.0	35 (8)	Health, FAO, USAID, AU/IBAR, UNOCHA

**Table 2:** Topic areas tested in exercises

**Location**	**Surveillance, information sharing**	**Disease prevention and control**	**Commun-ications**	**Medical surge capacity**	**Response coordination**	**Crisis manage-ment**
Cambodia	X	X	X	X		
China	X	X	X			
Lao PDR	X		X		X	
Myanmar	X	X				X
Thailand	X		X	X	X	
Vietnam	X				X	X
Palestine	X	X	X	X		
Jordan – sub-national	X	X	X	X	X	
Jordan – national	X	X	X	X		
Southeast Asia	X	X	X			
Middle East	X	X	X	X	X	X
East Africa	X	X	X			

Table 3 highlights the participant evaluation from six exercises that used questionnaires with Likert Scale questions. Participants who completed these evaluation forms consistently rated the overall quality of the exercises as high (88-100% rating the exercise as good or excellent) with one exception (Middle East sub-regional exercise 59% rating the exercise as good or excellent). Participants also consistently rated the exercises highly for helping them to understand the roles and responsibilities of organizations and agencies responding to an influenza pandemic (91-94% rating the exercise as good or excellent in this area) with one exception (China sub-regional exercise 76% rated the exercise as good or excellent in this area). Participants differed on what they felt about the quality of the information shared in the exercises (67%-93% rated the information as good or excellent). Participants rated the exercises lowest on their ability to identify key gaps in performance (50%-73% agreeing or strongly agreeing the exercises identified important key gaps). There are a variety of factors that could explain this result. First, tabletop exercises are discussion based and do not directly test operational capabilities. This may limit the ability of tabletop exercises to concretely identify key operational gaps. Second, tabletop exercises are subjective and participants may disagree about what constitutes a key gap. Third, the limited time frame to conduct tabletop exercises and the limited number of topics that can be discussed in that time frame may inhibit the ability of these exercises to identify a significant number of key gaps. A fourth possibility is that cultural sensitivities in some of these countries may have limited participants’ comfort in identifying gaps in their government’s preparedness system. The exercises were most successful at helping participants gain knowledge that they planned to use to improve the preparedness of their organization (82%-100% agreeing or strongly agreeing that they would use what they learned from the exercise).

**Table 3:** Participant likert scale ratings of exercises

**Question**	**China**	**Lao PDR**	**Myanmar**	Jordan – National*	**Palestine**	Middle East*
Overall quality of exercise (% good or excellent)	88	94	91	86	100	59
Quality of information exchanged (% good or excellent)	93	89	68	--	100	73
Key gaps identified (% agree or strongly agree)	50	77	73	66	68	--
Better understanding of roles (% agree or strongly agree)	76	100	91	--	94	91
Plan to use knowledge gained (% agree or strongly agree)	88	100	100	100	100	82

*Missing data (--) for the Jordan-National exercise and the Middle East exercise are due to the fact that these questions were not asked of exercise participants in those exercises.

Table 4 summarizes the qualitative feedback provided by participants in their evaluation forms. Three general themes came out of the participant comments on the most useful aspects of the exercises: the ability of exercises to raise awareness and understanding about public health threats, the ability of the exercises to assist in evaluating plans and identifying priorities for improvement and the ability of the exercises to build relationships and enhance preparedness and response capabilities across sectors and across countries in a geographic region. Participants also left the exercises with specific ideas about the most important follow-up actions that they should take in the near future. Specifically, participants identified better planning, improved planning coordination across sectors and countries and better training of health workers and response personnel. Finally participants provided feedback on the use of tabletop exercises for pandemic influenza preparedness. No participants stated that they felt the exercises involved too many sectors. In fact, many participants reported that they felt more sectors should be involved and that exercises should also involve more private sector partners and more partners from NGOs. Participants also felt that more could be done in the exercises to ground theoretical responses with more practical responses.

**Table 4:** Summary of participant qualitative feedback on exercises

**Question**	**Response summary**
What was most useful about the exercises?	● Helped to understand the extent of the threat
	● Raising awareness of challenges faced
	● Showed the importance of having solid plans in writing
	● Assisted in determining priorities
	● Demonstrated the importance of regional cooperation
	● Showed how multiple sectors of government should cooperate in planning and response
	● Improved confidence and team building
	● Sharing information and joint learning
	● Identification of potential solutions to challenges
What are the most important actions that should be taken based on the exercise?	● More frequent communications with partners
	● Development of better team preparations for response
	● Developing a detailed plan to follow up with gaps identified
	● Take planning more seriously and start working on it now
	● Delegate tasks better across all responsible parties
	● Significantly revise and improve existing plans
	● Better training of all health workers
	● Improve public awareness of the threat
	● Continue to meet regularly and assess improvements
	● Encourage more collaboration between animal and human health personnel
	● Create a regional rapid response team
	● Harmonize communication materials across all partners
What suggestions do you have to improve future exercises?	● Involve more private sector participants
	● Include more NGOs as participants
	● Better sharing of plans across all sectors and locations prior to the exercise
	● Hold more workshops and meetings prior to the exercises
	● Brief non-health sector participants better about disease etiology and transmission prior to the exercise
	● Do more to link theoretical responses to practical responses
	● Develop “train the trainer” approaches in the exercise
	● Provide more time to discuss each topic
	● Provide more opportunities for non-health sector participants to participate

Some health leaders who were part of exercise planning teams participated in semi-structured face-to-face interviews in January 2013. Countries that reported having pre-existing exercise programs prior to participating in the exercises described here were much more likely to report conducting exercises at regular intervals over time compared to countries that did not report a pre-existing exercise program. Most countries reported modifying and using some or all of the exercise template materials that were developed for the exercises described here. However, one country that had no prior exercise experience organized and carried out numerous sub-national exercises on their own after participating in the national and sub-regional exercise. Health leaders in this country reported that participating in an exercise helped to motivate them to develop an exercise program and regularly assess different aspects of their public health preparedness. The largest barriers to continued exercising that were reported included lack of financial resources and limited support among leadership to develop and sustain an exercise program.

## Conclusions

These experiences suggest that exercises can be a valuable, low-burden tool to improve emergency preparedness and response in countries around the world. They also demonstrate that countries can work together to develop and conduct successful exercises designed to improve regional preparedness to public health threats. Regular participation in exercises is associated with improved overall response to public health threats [26]. Countries that participated in sub-regional exercises together reported that these exercises improved their response to the 2009 H1N1 pandemic [24,27]. But exercises are not perfect. Research has called into question the ability of exercises to adequately expose operational and logistical gaps [28]. This is consistent with our finding that exercise participants rated the exercises lowest for identifying key gaps. In addition, there is a lack of consensus on what makes exercises effective tools to assess public health preparedness and how the outputs of exercises such as AARs can be used to support and improve public health preparedness efforts [29]. Thus, the development of standardized evaluation methods for exercises may be an additional tool to help focus the actions to be taken as a result of the exercise and to improve future exercises. Despite these flaws, exercises show great promise as tools to build relationships, assess performance and improve collaborative planning for public health threats across multiple sectors and multiple countries over time.

## Abbreviations

AAR: After action report; AU/IBAR: African Union/International Bureau for Animal Resources; FAO: Food and Agriculture Organization; NGO: Non-Governmental Organization; SARS: Severe acute respiratory syndrome; UNDP: United Nations Development Program; UNFPA: United Nations Population Fund; UNICEF: United Nations Children’s Fund; UNOCHA: United Nations Office for the Coordination for Humanitarian Affairs; UNRWA: United Nations Relief Works Agency; USAID: United States Agency for International Development.

## Competing interests

The authors declare that they have no competing interests.

## Authors’ contributions

DJD and MM designed and conducted the study. DJD led the writing and analysis. MM contributed substantially to both the analysis and writing. Both authors read and approved the final manuscript.
